# Eating Disorders and Dietary Supplements: A Review of the Science

**DOI:** 10.3390/nu15092076

**Published:** 2023-04-25

**Authors:** Susan J. Hewlings

**Affiliations:** Nutrasource Pharmaceutical and Nutraceutical Services, Inc., Guelph, ON N1G 0B4, Canada; sue.hewlings@gmail.com

**Keywords:** eating disorders, dietary supplements, disordered eating, eating disorder risk factors

## Abstract

Disordered eating is a serious health concern globally. The etiology is complex and multidimensional and differs somewhat for each specific eating disorder. Several risk factors have been identified which include psychological, genetic, biochemical, environmental, and sociocultural factors. Poor body image, low self-esteem, teasing, family dynamics, and exposure to media images have also been identified as risk factors. While it is enticing to consider a single behavioral risk factor, doing so fails to consider the documented environmental, social, psychological, biological, and cultural factors that contribute to the development of an eating disorder in a multidimensional and complex integration that is undoubtedly unique to everyone. Focusing only on any one factor without taking the complex etiology into account is remiss. For example, it has been suggested that the use of dietary supplements may lead to eating disorders, despite a lack of evidence to support this conjecture. Therefore, the purpose of this review is to examine the evidence-based risk factors for eating disorders and discuss why connecting dietary supplements to eating disorder etiology is not supported by the scientific literature and may interfere with treatment. Established, effective prevention and treatment approaches for eating disorders should be the focus of public health initiatives in this domain.

## 1. Introduction

Disordered eating is a challenging health concern that clinicians have been treating and researchers have been investigating for decades. A systematic review reported lifetime prevalence rates for anorexia nervosa of 1.4% (0.1–3.6%) for women and 0.2% (0–0.3%) for men, for bulimia nervosa of 1.9% (0.3–4.6%) for women and 0.6% (0.1–1.3%) for men, and for binge eating disorder of 2.8% (0.6–5.8%) for women and 1.0% (0.3–2.0%) for men [1]. Clinically diagnosable eating disorders are present in 1–3% of adolescents. Unfortunately, traditional treatment programs have demonstrated limited success [2]. Considering the prevalence, consequences, and limited success of treatment efforts, ongoing research interest in identifying risk factors with the goal of preventing and treating eating disorders has intensified.

Disordered eating attitudes range on a continuum from “normative discontent” to a diagnosed eating disorder. Eating disorders are often comorbid with mood and anxiety disorders, obsessive compulsive disorder, and other psychological conditions. The etiology is complex and multidimensional and differs somewhat for each specific eating disorder, as defined by the *Diagnostic and Statistical Manual of Mental Disorders* V (DSM-V) [3,4]. Eating disorders do not have a singular cause; they have a multidimensional etiology and complexity often with multiple comorbidities. However, multiple risk factors for developing an eating disorder have been suggested. These include psychological, genetic, biochemical, environmental, and sociocultural factors. Longitudinal research examining risk factors suggests that negative body image and disordered eating are the strongest predictors for the development of eating disorders in individuals of all ages [5]. Although there is a higher prevalence of eating disorders in women, men are also at risk and may be more likely to go undiagnosed [6]. Research has identified that the onset of disordered eating behaviors is connected to exposure to television, social media, and other environmental exposures as well as societal pressures to achieve a certain body ideal. This includes the uses of social media and photo editing [7]. While it may be enticing to focus only on a single behavior as a potential risk factor, doing so fails to take into account the documented environmental, social, psychological, biological, and cultural factors that contribute to the development of an eating disorder in a multidimensional and complex integration that is undoubtedly unique to each individual [8], thereby making efforts too focused on any one factor remiss. For example, it has been suggested that the use of dietary supplements may cause eating disorders, despite a lack of evidence to support this conjecture [9,10].

Therefore, this review examines the evidence-based risk factors for eating disorders, elucidates the lack of support in the scientific literature for dietary supplements as an etiologic factor in eating disorders, and, somewhat paradoxically, describes the beneficial role of dietary supplements in the treatment of eating disorders.

## 2. Known Risk Factors for Eating Disorders

A risk factor occurs before the associated outcome; it is a characteristic associated with an increased likelihood of the outcome being studied. It can be used to determine high-risk and low-risk subgroups [11]. It should not be confused with symptoms or behaviors associated with the disease itself. A risk factor is referred to as a causal risk factor if can be shown that when it is manipulated, it changes the probability of the outcome. It is very important to note that use of the word “causal” in this circumstance does not imply that the risk factor is the only cause of the outcome, nor does it address the pathways through which the causal risk factor might have its effect. It only means that manipulating the variable leads to a reduction in the incidence of the outcome [12].

Risk factors for developing an eating disorder are multidimensional and complex and often co-occur with the diagnosis of depression or anxiety. The literature indicates that eating disorder incidence is secondary to mental health conditions (e.g., depression, anxiety, and obsessive compulsive disorder) in this population. Other risk factors include poor body image, low self-esteem, previous trauma, environmental stress and pressures, real-world exposure (e.g., social media or other media), and several more [3,5,6,7,13,14,15]. In addition, it has been observed that populations such as sexual minority groups (e.g., those who identify as lesbian, gay, bisexual, questioning, queer, asexual, or those who feel their sexual orientation identity cannot be captured with existing terminology (LGBQA+) and those who report same-sex or same-gender attraction and/or behavior) are at a greater risk for eating disorder (ED) symptoms and behaviors. In addition to negative body image, those experiencing eating disorders are said to engage in one or more risky behaviors which may include: the use/abuse of laxatives, the use/abuse of prescription diuretics, the use of nicotine (e.g., cigarettes or vapes), exercise fixation, self-induced vomiting after eating, the maintenance of unrealistic beauty standards, irrational and maladaptive beliefs about body fat, and harsh self-evaluation and self-criticism [3,5,6,7,13,14,15]. These have been identified as primary risk factors that occur before an eating disorder and therefore can be considered in the design of prevention efforts. There is an extensive amount of literature regarding risk factors for eating disorders.. It is beyond the scope of this review to list them all, but the reader is referred to a recent umbrella review of meta analyses. This review incorporated 50 risk factors from nine meta-analyses and rated the strength of the evidence of these factors. There was no strong evidence behind any of the assessed risk factors. However, the strongest evidence was associated with childhood sexual abuse as a risk factor for bulimia and appearance-related teasing victimization for any eating disorder [16].

A 2021 clinical report published by the American Academy of Pediatrics (AAP) provides recommended questions a pediatrician should ask a patient with a suspected eating disorder as part of their initial screening and assessment. These recommendations do not include any questions about dietary supplement use. Rather, the report recommends questions surrounding mental health, prescription drug use, non-prescription drug use, stimulant use, laxative use, nicotine use, cigarettes, vaping, etc. (the reader is referred to Table 2 in Identification and Management of Eating Disorders in Children and Adolescents, American Academy of Pediatrics Clinical Report) [17]. The AAP therefore did not identify dietary supplements as a risk factor in its screening for eating disorders.

According to the DSM-V, dependence on enteral feeding or nutritional supplements taken orally is a symptom or warning sign of an eating disorder, specifically Avoidant/Restrictive Food Intake Disorder (ARFID) [4]. This should not be confused with a risk factor; as mentioned above, a risk factor occurs before the behavior [11]. It is reasonable to state that the abuse, not use, of some supplements may be a sign or symptom of an eating disorder, not a risk factor.

## 3. Triggers

According to major professional organizations such as the Academy of Nutrition and Dietetics (the Academy), a key aspect of the prevention and treatment of eating disorders is to identify triggers, which have significant interindividual differences. A trigger in this context is defined as “an antecedent that generates or provokes an eating disordered behavior as a coping mechanism” [18]. The Academy’s guidance goes on to state “Each eating disorder client will have unique triggers that are specific to their underlying psychopathology. The nutrient composition of food, environmental variables such as location and smell, and dysfunctional relationships with people are common triggers”. Dietary supplements are not mentioned as a trigger in the guidance. However, the Academy recognizes the importance of dietary supplements as part of the nutrition plan of care (Section 3.13A) [18]. It should also be noted that among those with an eating disorder, the most frequently used or abused substances were alcohol, laxatives, emetics, diuretics, amphetamines/stimulants, and other drugs/substances (i.e., not dietary supplements) [19,20,21].

## 4. Dietary Supplement Use in Adolescents

Monitoring the Future (MTF) is an epidemiological study conducted by researchers from the University of Michigan’s Institute for Social Research, and since 1975, it has assessed 12th grade students, and since the 1990s, 8th and 10th grade students as well. It provides research-driven information regarding problem behaviors of the use of illegal drugs, alcohol, and tobacco, and the non-prescription use of psychotherapeutic drugs. The report states the following relative to diet pills: “Use of “diet pills”, which are over-the-counter stimulants, were at the lowest level ever recorded by the survey in 2022 for lifetime, past 12-month, and past 30-day use. Today’s levels of past 12-month use are more than five times lower than their peak of 21% in 1982, when “diet pills” were first included on the survey. After 1982, prevalence fell quickly over the next ten years to 8% in 1993; this was a particularly positive development because nearly all these “diet pills” contained phenylpropanolamine (PPA), which was marketed as an over-the-counter medication, not as a dietary supplement. The Food and Drug Administration has since determined PPA has health risks for the user and in 2005 removed PPA from over-the-counter sale. Use stabilized through the mid-1990s at around 9.4%, rose after 1998 to reach 15.1% in 2002, and then declined to today’s low of 1.6%”. This longitudinal study suggests a marked decline in the use of “diet pills” by high-school-aged students [22].

The MTF report also provides information about some dietary supplements. For example, it reports on creatine use. The report states the following: “Creatine is not a hormone or a drug, but a nutrient found in the skeletal muscle of most animals. It is used to reduce the recovery time of muscles, to increase muscle mass, and to thereby enhance performance for high-intensity, short-duration exercises”. There was a significant increase in use from 2021 to 2022, especially in 10th and 12th graders. The researchers suggest that this increase was due to an increase in adolescents involved in weight training and fitness endeavors during the pandemic; the increased use was not for weight loss purposes [22].

Data from the National Health and Nutrition Examination Survey (NHANES) of 2017–2018 were used in a 2020 publication to estimate the prevalence of dietary supplement use among U.S. children and adolescents <19 years of age. The data were categorized as “takes a single dietary supplement” or “takes two or more dietary supplements” and then categorized by specific dietary supplement product types. The data indicated that approximately one-third of all children and adolescents took a single dietary supplement at least once in the 30 days leading up to the survey. In adolescents, the most frequently used dietary supplement was a multivitamin mineral (17.3%); Vitamin D was the second most consumed dietary supplement (5.4%), followed by Vitamin C (4.2%). Weight loss or weight gain supplements were not identified as supplements used most often by adolescents [23]. A study using the NHANES 1999–2016 data aimed to characterize types and trends of dietary supplements, especially non-vitamin/non-mineral dietary (NVNM) products, commonly consumed by U.S. children along with motivations for their use. NVNM products included omega-3 fats, probiotics, botanicals, protein, amino acids, carnitine, caffeine, etc. The researchers reported that approximately 68% of children do not use dietary supplements at all; 4% reported using NVNM supplements. Omega-3 was the most used NVNM supplement, followed by probiotics. The two most common motivations for use were “to maintain health” and “to improve overall health” [24]. Weight loss as a motivation to use supplements and weight loss supplements were not reported by this cohort. Taken collectively, these analyses of the NHANES data suggest that dietary supplements for weight management or muscle growth are not used by a significant portion of teens.

A study of 348 athletes aged 15–18 years from 4 countries participating in 18 sports was conducted to assess their knowledge and attitudes towards supplements. Protein was the most widely consumed supplement, with 54.5% of the athletes reporting use of protein supplements. The major motivation for consuming supplements was to enhance athletic performance (34.5%) followed by health improvement (27%) and recovery (25.9%), with only (10.9%) reporting physical appearance as a motivating factor. The males used significantly more whey protein, creatine, amino acids, caffeine, and nitrous oxide compared to females, who took more vitamins and mineral complexes, while there was an almost equal use of energy drinks, glutamine, and carbohydrates between sexes [25]. The results of this study are supported by a survey of 567 Canadian athletes aged 11–25 years who report performance gains as the primary motivation for the use of dietary supplements such as protein, creatine, and amino acids [26].

A cross-sectional study including 6212 girls and 4237 boys between the ages of 12 and 18 years was conducted to determine the prevalence and correlates of products used to improve weight and shape. The authors concluded that the use of supplements to improve weight and shape was rare and more common in males than females. They also reported that the products being used, such as protein and creatine, were being used to gain weight and muscle, not to lose weight [27]. While any attempt to change one’s body can be viewed as body dissatisfaction, engaging in exercise training and consuming protein and other non-vitamin/mineral supplements for increased muscle mass is also recommended as a means to enhance health and fitness as well as to optimize performance and recovery in sports [28,29,30,31].

In summary, the use of “diet pills” by adolescents appears to have decreased markedly since 1982 [22]. Furthermore, the major motivations for dietary supplement use in adolescents are to maintain or improve overall health, as well as for positive performance gains, as opposed to diet and weight loss [27,28,29,30,31,32,33,34,35].

## 5. Studies that Attempt to Link Dietary Supplements to Eating Disorders

Activist organizations have cited studies alleging a connection between dietary supplements and eating disorders [36,37]. These studies are characterized by faulty designs with conclusions based on unsupported data. Furthermore, there is not a correct or common definition of “dietary supplement” or “diet pills” used or applied to the referenced studies or to the proposed legislation. This is concerning, as such studies are being used to influence policy and support legislation to reduce access to healthy nutrients for consumers.

A prospective study was conducted over a 15-year period to examine the association of “diet pill” and laxative use for weight control with women with eating disorders. It should be noted that the study did not clearly define “diet pill”, and laxatives were not classified as dietary supplements but rather as over-the-counter medications. In their introduction, the authors state that “an estimated 15% of adults report lifetime use of “diet pills” for weight loss. Lifetime use of laxatives for weight control among adults is estimated at 5% and from 15% to 62% in those with eating disorders” [32]. It should be noted that the reference used to support this statement was based on a non-validated survey—the determination of whether one used “diet pills” was made from the following question: “We would like you to tell us about any other products that were NOT prescribed by a doctor that you have taken to control your weight, including over-the-counter products such as pills, powders, and liquids. This includes dietary supplements and natural or herbal weight loss aids not prescribed by a doctor. Have you ever taken any of these products to control your weight?” The response categories for all questions were “Yes, in the past 12 months,” “Yes, but not in the past 12 months,” and “No” [33]. The question failed to define a dietary supplement and did not differentiate them from over-the-counter medications (e.g., typical laxatives and diuretics). Therefore, the amount of dietary supplement use by participants could not be determined in this study.

Setting aside this methodological flaw in the survey question, results from this study also have been wrongly cited as evidence of a causal relationship between “diet pills” and eating disorders, but that causality was not demonstrated either. The survey purported a correlation between individuals who reported using products to control their weight and a subsequent diagnosis of an eating disorder. However, the association cannot be characterized as causal, or even a contributing factor, to the subsequent diagnosis. Without further elucidation, the eating disorder may have led to the use of “diet pills”. This study, along with a similar study [34], are often cited by some as evidence of a prospective causal link between products used for weight control and future eating disorder diagnosis. However, it should be noted that the DSM-V makes no mention of dietary supplements in its description of risk factors or diagnostic criteria [4]. While further investigation is warranted to determine whether dietary supplement abuse is a valid screening criteria for diagnosing an eating disorder, neither of these studies support a causal nature of the relationship.

Separately, a cross-sectional study of university students aged 18–26 was conducted to explore the connection between ergogenic supplement use and disordered eating attitudes and behaviors and to compare differences by sex. A total of 1633 university undergraduate students from 10 top-ranked National College Athletics Association (NCAA) Division I colleges completed a non-validated online survey on supplement use, athletic activities, and eating attitudes and behaviors. A major design flaw in this study is that illegal substances and prescription drugs such as anabolic steroids, human growth hormone, and androstenedione were grouped together with dietary supplements. These are not dietary supplements and are not sold under this regulatory classification. The researchers noted males reported higher supplement use than females. They also reported that female supplement users had higher scores on eating disorder surveys than non-users. This outcome, of course, is not an indication of a cause-and-effect relationship but rather an association. As with the previous study, the dietary supplement use may have resulted from, rather than being a cause of, the eating disorder. Information from this study should be considered in light of its multiple limitations, including the use of a non-validated survey as well as the misclassification of dietary supplements [9]. Another study by the same team also included non-dietary supplement use in the assessment and suggested that dietary supplements were laced with harmful substances without testing the supplements the participants were consuming to determine if they were actually adulterated [10].

A cross-sectional study of 6212 girls and 4237 boys aged 12–18 years was conducted from 1996 to 1999 to explore the prevalence and correlates of products used to improve weight and shape among male and female adolescents. An adapted version of the McKnight Risk Factor Survey (MRFS) was used for assessment. Participants were asked, “During the past year, how often did you use any of the following products because you thought they would improve physical appearance or help you gain weight, strength, or muscle mass?” They then were asked how often (never, less than monthly, monthly, weekly, or daily) they used each of the following seven products: protein powder or shakes, creatine, weight loss shakes/drinks, amino acids/hydroxy methylbutyrate (HMB), DHEA, growth hormone, and anabolic/injectable steroids. They reported that “approximately 4.7% of the boys and 1.6% of the girls used protein powder or shakes, creatine, amino acids/HMB, dehydroepiandrosterone, growth hormone, or anabolic/injectable steroids at least weekly to improve appearance or strength”. The authors concluded that “…use of potentially unhealthful products that are perceived to improve appearance, muscle mass, or strength is relatively rare and more common among male than female individuals”. They interjected their own biases without providing evidence that the products were “unhealthful”, and they continued to say that the use of said products is harmful and leads to unhealthful weight control methods, which contradicts their own results that indicate use is rare and the motivation for use was primarily to increase muscle mass and strength rather than lose weight. However, major concerns surrounding the design of this study include that the data were collected more than 20 years ago, the researchers did not test the validity of their adapted survey, and they grouped dietary supplements such as protein powder and creatine with illegal drugs such as growth hormone and injectable anabolic steroids [27]. The study had several limitations, and the authors’ conclusions are highly misleading.

In the review article entitled “The Dangerous Mix of Adolescents and Dietary Supplements for Weight Loss and Muscle Building Legal Strategies for State Action”, the authors attempted to provide a rationale for state legislation to restrict access to dietary supplements, particularly for adolescents, by providing alleged evidence of dangers of weight loss and muscle-building supplements. They stated that health care providers do not recommend the use of dietary supplements despite countless references stating otherwise [35]. They failed to recognize that dietary supplements for weight gain are in fact recognized by major professional organizations to prevent and treat many conditions including sarcopenia, frailty, and eating disorders [18,28,38]. Furthermore, supplements such as creatine have been shown to be safe and effective in children and teens and are often used to manage several clinical concerns, including inborn errors of metabolism [39]. For a more in-depth review of this topic, see Jagim et al. 2021 [40]. It is concerning that these articles refer to dietary supplements, laxatives, and illegal anabolic steroids as similar products [27,35,41].

A retrospective observational study examining adverse events reported between January 2004 and April 2015 in the Food and Drug Administration Adverse Event Reporting System (AERS) on food and dietary supplements database was published by Or et al. in 2019. The authors attempted to quantify relative risk for “severe medical events” by type of dietary supplement among individuals 0 to 25 years. They included 977 adverse event reports in their analysis. They categorized the dietary supplements based on a non-validated web search approach using only six categories: weight loss, colon cleanse, muscle building, sexual function, vitamin, or other. It should be noted that this omits many supplement categories such as cognitive function, joint health, digestive health, and more. Furthermore, the categorization was based on subjective perceptions of claims found on the internet, not necessarily reflective of the intent of use by the consumer. The authors reported that “Among adults aged 18–25 years, the frequency of adverse events seemed to be the highest for weight-loss supplements in both women and men. Within the 18–25 years group, adverse events associated with supplements sold for muscle building, cleanse, sexual function, and energy seem to be more commonly reported among men compared with women”. It is unknown what statistical values support “seem to be”. It should be noted that vitamins were also associated with adverse events. This evaluation is flawed, as the underlying data set from FDA that the authors accessed contains AERs that are self-reported and based on associations, often not causal, and therefore, the consumption of the supplement may not be related to the adverse event at all [42].

When discussing AERS for young children, it should be noted that the majority are related to unintentional ingestions. In fact, in children aged 1–5 years, “Unintentional Ingestion with Symptoms” represented 82% of the group [43]. When they are removed from the AERs, the overall number is much lower, especially when considering the total number of supplements ingested without any incidence during that same 12-year period.

## 6. Dietary Supplements in Eating Disorder Treatment

Dietary supplements, as a category, are sometimes falsely dismissed as being beyond the oversight of the FDA and without demonstrated benefit. However, this ignores that dietary supplements, which are subject to regulation by the FDA, are routinely used in the treatment of eating disorders as well. It is standard of care to utilize dietary supplements as part of a multidimensional treatment plan for eating disorders. The Academy of Nutrition and Dietetics notes in their Revised 2020 Standards of Practice for the Professional Practice of the Registered Dietitian that “dietary supplements, ranging from multivitamins, to botanicals, to protein supplements, to calorie-protein supplements and more are standards of care for those being treated for an eating disorder”. Dietary supplements are used as part of recovery for an individual to support health and recovery [18].

## 7. Successful Prevention Programs

When determining actions designed to result in health behavior change, it is critical to assess what has been reported in the scientific literature to be effective. While providing a comprehensive discussion of the eating disorder prevention literature is outside the context of this review, it is important to point out a few relevant successful efforts. Eating disorder prevention efforts have made documented progress in interventions that have been shown to reduce eating disorder symptomatology and risk of future eating disorders by successfully translating eating disorder risk factor research [44,45,46]. One of the most supported approaches is dissonance-based eating disorder prevention programs, referred to as the Body Project. This initiative included several randomized (efficacy, effectiveness, and comparative) trials and rigorous tests of the intervention theory carried out by multiple independent research groups [45,47,48]. This prevention approach is based on the social psychological principle of cognitive-dissonance theory [49]. A recent meta-analysis of prevention programs identified 15 trials involving 5080 participants that tested whether eating disorder prevention programs significantly improved outcomes. Healthy lifestyle modification prevention programs, dissonance-based prevention programs, and a self-esteem/self-efficacy prevention program significantly reduced the future onset of eating disorders. Psychoeducational, cognitive behavioral, behavioral weight gain, interpersonal, and family-therapy-based prevention programs did not significantly reduce the future onset of eating disorders. Lifestyle modification and dissonance-based prevention programs significantly reduced the future onset of eating disorders in multiple trials, producing a 54% to 77% reduction in future eating disorder onset [50].

Third-wave behavioral interventions such as Acceptance and Commitment Therapy, Dialectical Behavior Therapy, Compassion-Focused Therapy, Functional Analytic Therapy, Schema Therapy, Metacognitive Therapy, and Mindfulness-Based Cognitive Therapy or Mindfulness-Based Stress Reduction are being used for prevention efforts in other mental health conditions. The evidence specific to the use of these interventions for eating disorders is growing. These interventions are based on changing cognitive processes, not cognitive content. A recent meta-analysis examined whether third-wave behavioral interventions are effective eating disorder prevention programs by assessing their effects on eating disorder risk factors in individuals that did not have an eating disorder. The results included 24 studies and indicated that third-wave behavioral interventions show potential as effective eating disorder prevention programs. These interventions led to modest improvements in eating disorder risk factors [51].

## 8. Discussion

Organizations advocating for policies to limit the ability of young people to purchase dietary supplements have stated that dietary supplements independently increase the risk of developing an eating disorder [37]. This conjecture contradicts the evidence base and undermines the critical need for prevention and treatment strategies driven by evidence-based guidelines. Those in support of these policies have stated dietary supplement use is a trigger for eating disorders, despite a lack of evidence to support their hypothesis, and in contrast to positions held by authoritative health organizations. Several of the statements that these groups have released to support their initiatives have led to the circulation of false information related to the causes and risk factors of eating disorders.

It is well documented that eating disorders are a significant health concern and it is important to have policies in place to address the issue. However, these policies must be driven by evidence-based, scientifically sound data. Health policy must also be carefully evaluated in the context of a complete understanding of the multi-dimensional complexity of the disease. It has been described that, “…currently, US federal, state, and county or city public health surveillance and evaluation systems gather very little if any data related to eating disorders, disordered weight-related behaviors, or risk factors for or sequelae of eating disorders” [41]. Basing policy recommendations on poorly designed research, which includes non-validated surveys that do not accurately classify dietary supplements, is problematic and irresponsible. This could lead to the allocation of limited resources and attention to a single issue that is ill-defined and has been improperly evaluated. It has been correctly stated that “…federal, state, and local policies and health care practices exacerbate eating disorders and disordered eating behaviors through either neglect or noxious effects of insufficiently evaluated programs and interventions” [41]. Furthermore, focusing on this single issue with little to no scientific support is harmful and distracts from efforts that would otherwise support properly informed interventions. Restricting dietary supplement access is incongruent with the advice of authoritative health professional organizations that play a key role in eating disorder treatment guidelines. One of the greatest concerns related to restricting access to dietary supplements of any type is that they support health for many individuals and are often included as part of care plans for many conditions.

## 9. Conclusions

The evidence to date does not support a causative role for dietary supplements in eating disorders. The use of dietary supplements for weight management in both male and female teens appears to be declining, and the objective of weight loss is not observed as a common motivation for the use of dietary supplements among this age group. There is some evidence to suggest a potential link between the use of some products and a later eating disorder diagnosis. However, this appears to be more of a symptom or associated behavior potentially valuable as a screening tool as opposed to demonstrating causality [11]. Therefore, it may be reasonable to state that the abuse, not use, of certain supplements may be associated with an eating disorder rather than being a risk factor.

However, there are established prevention and treatment programs for the disorder which trained health care professionals can utilize. Attention to the complex genetic, environmental, psychological, and cultural factors already identified may be useful to specific treatment approaches. Public health policy and resources should be focused on scientifically established approaches to reduce the prevalence of eating disorders.
